# Note from the Editors: Toxicogenomics Update

**Published:** 2005-02

**Authors:** Thomas J. Goehl, Kenneth S. Ramos

**Affiliations:** Editor-in-Chief, *EHP,* NIEHS, Research Triangle Park, North Carolina, E-mail: goehl@niehs.nih.gov; Toxicogenomics Editor, *EHP* ,University of Louisville Health Sciences Center, Louisville, Kentucky

*EHP* is continually evolving to meet the needs of our readership. Our intention in publishing a separate section in toxicogenomics for the past 2 years has been to feature research in this emerging field and to use our news articles as an educational tool to explain basic toxicogenomics principles to our general readership.

Feedback from our readership and the editorial board has indicated that our current coverage of the field of toxicogenomics might not be achieving the intended goals. After careful deliberations with stakeholders, the editors have decided that a better way to meet the needs of our readership and the field of toxicogenomics is to incorporate the Toxicogenomics section into the monthly issues. This change will be implemented beginning this month.

The major advantage of this approach is that we can print toxicogenomics articles shortly after acceptance rather than holding them for publication in a quarterly section. In addition, published articles will gain wider exposure among the general readership, helping to better fold toxicogenomics into the environmental health research portfolio. Another advantage is that we will able to more efficiently achieve our educational goals by making the field more accessible to our general readership.

What does the change mean for coverage?

- A toxicogenomics research section will be included within each monthly issue when research papers are available.
- *EHP* will continue news coverage through a variety of formats including updates, investigative articles, and policy discussions as warranted.
- To meet our educational goals, we have compiled all toxicogenomics feature articles (Focus articles) in a Toxicogenomics Primer that will be placed on a CD to be included with the March issue and made available at various toxicogenomics meetings.
- We will incorporate the toxicogenomics calendar, fellowships and grants, new books, and book reviews within the monthly sections.
- We are working to enhance the Toxicogenomics section of the *EHP* website by adding a compilation of news and research articles, “Notes from the Field,” and Resources.
- Editorials on toxicogenomics will continue to be solicited.

We look forward to your continued support of *EHP* and the Toxicogenomics section.

